# Event-Free Survival in Patients with Early HER2-Positive Breast Cancer with a Pathological Complete Response after HER2-Targeted Therapy: A Pooled Analysis

**DOI:** 10.3390/cancers14205051

**Published:** 2022-10-15

**Authors:** Sandra M. Swain, Harrison Macharia, Javier Cortes, Chau Dang, Luca Gianni, Sara A. Hurvitz, Christian Jackisch, Andreas Schneeweiss, Dennis Slamon, Pinuccia Valagussa, Yolande du Toit, Dominik Heinzmann, Adam Knott, Chunyan Song, Patricia Cortazar

**Affiliations:** 1 Lombardi Comprehensive Cancer Center, Georgetown University Medical Center, MedStar Health, Washington, DC 20057, USA; 2 F. Hoffmann-La Roche Ltd., 4070 Basel, Switzerland; 3 Quirónsalud Group, IOB Institute of Oncology, Madrid and Barcelona, 08023 Barcelona, Spain; 4 Vall d’Hebron Institute of Oncology (VHIO), 08023 Barcelona, Spain; 5 Department of Medicine, Breast Medicine Service, Memorial Sloan Kettering Cancer Center, New York, NY 10013, USA; 6 Fondazione Michelangelo, 20121 Milano, Italy; 7 David Geffen School of Medicine, University of California Los Angeles, Los Angeles, CA 94720, USA; 8 Sana Klinikum Offenbach, 63069 Offenbach, Germany; 9 National Center for Tumor Diseases (NCT), 69120 Heidelberg, Germany; 10 Genentech, Inc., South San Francisco, CA 94080, USA

**Keywords:** dual HER2 targeting, early breast cancer, event-free survival, HER2, pathologic complete response, pertuzumab, trastuzumab

## Abstract

**Simple Summary:**

The current standard of care for patients with HER2-positive early breast cancer who have a pathological complete response after neoadjuvant HER2-targeted therapy plus chemotherapy is continuation of HER2-targeted therapy in the adjuvant setting. However, it is not clear how long-term outcomes differ by the HER2-targeted regimen received in each setting. To investigate this question, we pooled patient-level data (n = 1763) from neoadjuvant studies of trastuzumab and pertuzumab to evaluate outcomes with respect to single versus dual HER2 targeting in the neoadjuvant and adjuvant settings. Patients treated with dual HER2-targeted therapy in both the neoadjuvant and adjuvant settings had the highest 4-year event-free survival rates, suggesting that this treatment approach may provide the most benefit for patients with HER2-positive early breast cancer.

**Abstract:**

The standard-of-care for patients with pathological complete response (pCR) after neoadjuvant human epidermal growth factor receptor 2 (HER2)-targeted therapy plus chemotherapy is continuation of HER2-targeted therapy in the adjuvant setting. Our objective was to evaluate risk of recurrence or death in these patients and determine if outcomes differed by the HER2-targeted regimen received in each setting. We analyzed patient-level data from five randomized trials evaluating trastuzumab, pertuzumab, or both as part of systemic neoadjuvant and adjuvant therapy for HER2-positive early breast cancer, and assessed event-free survival (EFS) in 1763 patients. Patients with pCR had decreased risk of an EFS event versus those with residual disease (unadjusted hazard ratio [HR] = 0.35; 95% confidence interval [CI]: 0.27–0.46). Regardless of pCR status, after adjusting for baseline factors, reduction in EFS event risk was greater in patients administered pertuzumab/trastuzumab in both settings versus those administered only trastuzumab in both settings (HR = 0.36; 95% CI: 0.26–0.49), or pertuzumab/trastuzumab in the neoadjuvant setting and only trastuzumab in the adjuvant setting (HR = 0.67; 95% CI: 0.47–0.96). Patients with pCR had longer EFS than those with residual disease. Patients treated with pertuzumab/trastuzumab in both the neoadjuvant and adjuvant settings had the lowest risk of breast cancer recurrence.

## 1. Introduction

Patients with human epidermal growth factor receptor 2 (HER2)-positive early breast cancer (EBC) who have a pathological complete response (pCR) after neoadjuvant HER2-targeted therapy in combination with chemotherapy have a lower risk of recurrence and death compared to patients with residual invasive disease at surgery [1,2,3,4,5,6,7]. However, a substantial proportion of these patients eventually experience disease recurrence, and recurrence is markedly increased in patients who do not receive HER2-targeted therapy [4]. Thus, the current standard of care for patients with HER2-positive EBC who have a pCR after HER2-targeted therapy plus chemotherapy in the neoadjuvant setting is continuation of HER2-targeted therapy in the adjuvant setting [8,9,10]. The current standard of care for patients with residual disease after neoadjuvant HER2-targeted therapy and chemotherapy is adjuvant trastuzumab emtansine (T-DM1) [10,11,12], which has substantially improved outcomes for these patients [13].

Large studies have evaluated various HER2-targeted treatments as part of systemic (neo)adjuvant regimens to treat HER2-positive EBC, including single-antibody treatment with neoadjuvant trastuzumab followed by adjuvant trastuzumab, and dual-antibody treatment with neoadjuvant pertuzumab and trastuzumab followed by adjuvant trastuzumab with or without pertuzumab [5,14,15,16,17,18,19,20,21]. However, none of these studies assessed the effects of single versus dual HER2-targeted therapy after neoadjuvant therapy on long-term outcomes.

The NEOSPHERE trial included a comparison of chemotherapy plus single (trastuzumab) versus dual (pertuzumab plus trastuzumab) HER2-targeted therapy in the neoadjuvant setting. The pCR rate was significantly higher in patients who received the dual therapy [14]. Five-year progression-free survival (PFS) and disease-free survival (DFS) were also longer in this group; however, the confidence intervals overlapped, and the study was not powered to detect differences in PFS and DFS [17]. The APHINITY trial compared chemotherapy plus single (trastuzumab) versus dual (pertuzumab plus trastuzumab) HER2-targeted therapy in the adjuvant setting [22,23]. Three-year invasive disease-free survival (IDFS) was significantly improved with the addition of pertuzumab in patients with lymph node–positive disease [22]; with a larger difference between the treatment groups in this population at the 6-year analysis [23], and regardless of hormone receptor status [23]. While the results from these trials suggest improved outcomes with pertuzumab plus trastuzumab compared with trastuzumab alone in the neoadjuvant or adjuvant settings, data are lacking on whether dual versus single HER2-targeted therapy in the adjuvant setting after neoadjuvant treatment affect long-term outcomes.

To better understand the risk of recurrence or death in patients with HER2-positive EBC attaining a pCR after neoadjuvant systemic anti-HER2 therapy, we pooled patient-level data from neoadjuvant studies of trastuzumab and pertuzumab to evaluate outcomes with respect to single versus dual HER2 targeting in the neoadjuvant and adjuvant settings.

## 2. Materials and Methods

We pooled patient-level data from five randomized neoadjuvant trials in patients with HER2-positive EBC (including those with inflammatory breast cancer) who received trastuzumab, pertuzumab, or both as part of a systemic neoadjuvant regimen and for whom individual patient-level data were available. The following studies were included: HannaH (NCT00950300) [15,21], NeoSphere (NCT00545688) [14,17], TRYPHAENA (NCT00976989) [5,16], BERENICE (NCT02132949) [19,24], and KRISTINE (NCT02131064) [18,20]. All studies were conducted in accordance with Good Clinical Practice guidelines and the Declaration of Helsinki. Study protocols were approved by the institutional review board and/or ethics committee at each site. All patients provided written informed consent. The treatment arms in each study are shown in Table S1. All studies had individual patient-level data available for disease characteristics, pCR, and event-free survival (EFS). The number of overall survival events was too low to perform a robust analysis. While the chemotherapy backbones differed, groups were constructed based on the HER2-targeted therapy received in the neoadjuvant and adjuvant settings to create three groups: (1) those receiving trastuzumab in both the neoadjuvant and adjuvant settings (i.e., H→H); (2) those receiving pertuzumab plus trastuzumab in the neoadjuvant setting and trastuzumab in the adjuvant setting (i.e., PH→H); (3) those receiving pertuzumab plus trastuzumab in both the neoadjuvant and adjuvant settings (i.e., PH→PH) (see Table S1).

The primary objectives of the analysis were to compare EFS outcomes in patients who had a pCR and in those with residual disease and to determine if these outcomes differed by the HER2-targeted regimen received in the neoadjuvant and adjuvant settings. The secondary objective was to determine if these outcomes were influenced by disease characteristics such as clinical stage, nodal status, and hormone receptor status.

A pCR was defined as the absence of residual invasive cancer in the resected breast specimen and in the axillary lymph nodes (ypT0/Tis ypN0) after neoadjuvant systemic therapy. EFS was defined as the time from the date of randomization/enrollment (which occurred after initial diagnosis) to the date of disease recurrence or progression (local, regional, distant, or contralateral) or death due to any cause. The risk of recurrence or death was analyzed by HER2-targeted therapy received in the neoadjuvant and adjuvant settings (i.e., H→H; PH→H; PH→PH). Four-year EFS rates were estimated using the Kaplan–Meier method. Patients without an event were censored at the date of the last disease status assessment or of the last recorded visit for the patient. If no post-baseline tumor assessment was available, EFS was censored at the date of randomization. Average treatment effects were evaluated after adjusting for baseline hormone receptor status (negative, positive), clinical stage (stage I, II, and III), and age group (<40 years, 40−65 years, and >65 years). A weighted Cox model based on inverse probability of treatment weighting (IPTW) using propensity scores was applied to each pairwise comparison such that a weight was calculated for each patient that was equal to the inverse of the probability of receiving the treatment actually received, while accounting for imbalances in baseline factors (i.e., hormone receptor status, clinical stage, age group). Nodal status was not included as a separate variable since it is part of the clinical stage variable that takes into account tumor size, nodal status, and presence of metastases. Using the method of Hajage and colleagues [25], these weights were then incorporated into the Cox model to minimize the potential confounding effects of these variables. The propensity model was estimated using a logistic regression model. Absolute standardized mean differences in the key baseline factors were examined before and after applying the IPTW method. This analysis indicated that the covariates were well balanced after applying IPTW (Figure S1). Analyses were performed with R software (Vienna, Austria).

## 3. Results

### 3.1. Patient Populations

A total of 1763 patients were included in the analysis. Median follow-up was 71.6 months in the H→H group, 61.3 months in the PH→H group, and 62.4 months in the PH→PH group. There were some imbalances in baseline characteristics (Table 1). There were more patients with clinical stage II disease (71.5%) and hormone receptor–positive disease (60.5%) in the PH→PH group compared with the PH→H (46.5% and 49.0%, respectively) and the H→H (40.5% and 52.9%, respectively) groups.

The pCR rate among all patients was 43.8%. The pCR rate differed by tumor stage, clinical stage, hormone receptor status, and treatment modality (Table 2). The pCR rate was highest in the PH→PH group (56.7%), followed by the PH→H group (42.1%) and the H→H group (33.6%).

### 3.2. Event-Free Survival

**Figure 1:**
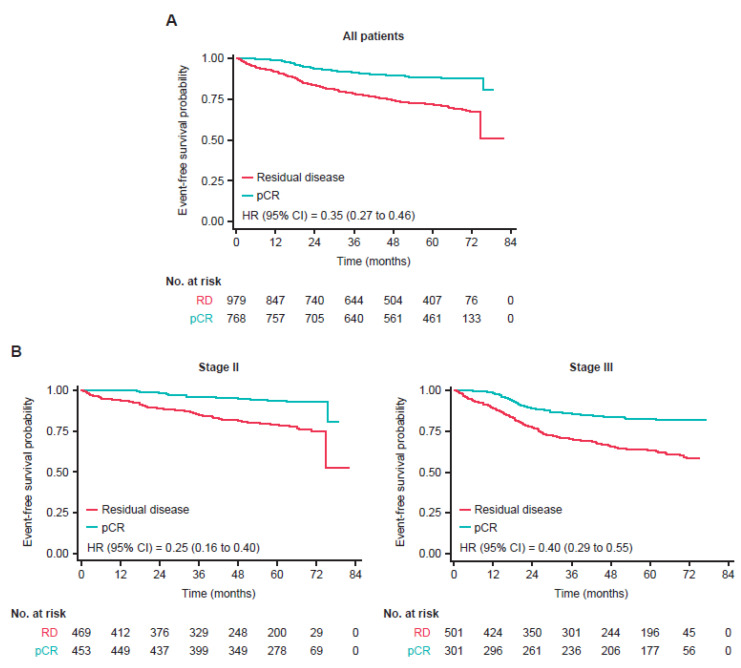

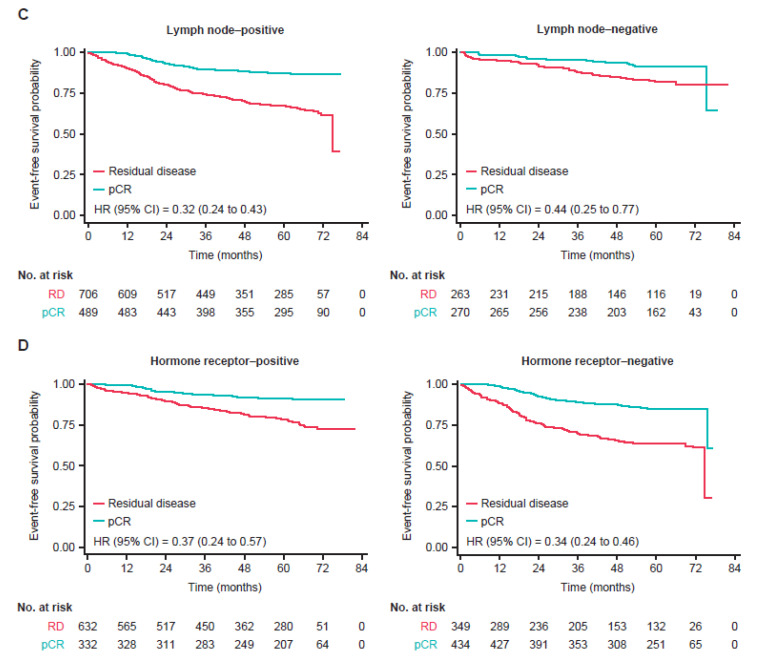
Event-free survival in patients with pCR after neoadjuvant systemic therapy (**A**) and by (**B**) clinical stage, (**C**) nodal status, and (**D**) hormone receptor status. The inverse probability of treatment weighting method was used to account for imbalances in the baseline factors of hormone receptor status, clinical stage, and age group. Abbreviations: CI, confidence interval; HR, hazard ratio; RD, residual disease.

**Figure 2:**
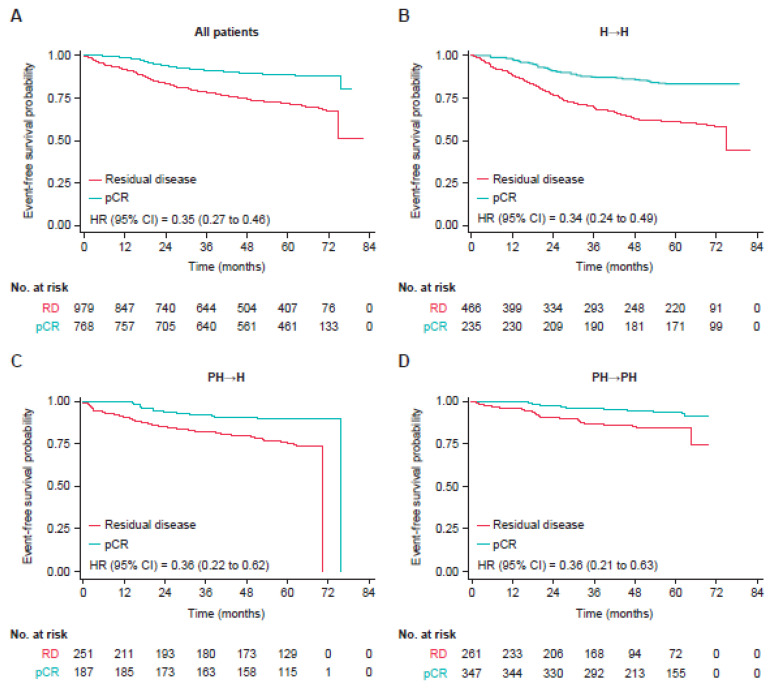
Event-free survival in patients with pCR after neoadjuvant systemic therapy (**A**) and in patients treated with trastuzumab in the neoadjuvant setting followed by trastuzumab in the adjuvant setting (H→H; (**B**)), in patients treated with pertuzumab plus trastuzumab in the neoadjuvant setting followed by trastuzumab in the adjuvant setting (PH→H; (**C**)), and in patients treated with pertuzumab plus trastuzumab in the neoadjuvant setting followed by pertuzumab plus trastuzumab in the adjuvant setting (PH→PH; (**D**)). The inverse probability of treatment weighting method was used to account for imbalances in the baseline factors of hormone receptor status, clinical stage, and age group.

Overall, patients with a pCR had a 65% reduction in risk of an EFS event compared to those with residual disease (unadjusted hazard ratio [HR] = 0.35; 95% confidence interval [CI]: 0.27–0.46]). Risk of recurrence was markedly decreased in patients who had a pCR compared to those with residual disease, irrespective of clinical stage, nodal status, hormone receptor status (Figure 1), or treatment modality (i.e., H→H; PH→H; PH→PH) (Figure 2).

In the pooled population of patients with and without a pCR, EFS varied by treatment modality (Table 3). Fewer patients in the PH→PH group (8.5%) had an EFS event than those in the PH→H group (18.0%) or the H→H (30.9%) group. There was a 44% reduction in the risk of an EFS event with PH→H compared with H→H (HR = 0.56; 95% CI: 0.43–0.73), a 64% reduction in risk of an EFS event with PH→PH compared with H→H (HR = 0.36; 95% CI: 0.26–0.49) and a 33% reduction in the risk of an EFS event with PH→PH compared with PH→H (HR = 0.67; 95% CI: 0.47–0.96).

Among patients with a pCR, fewer patients in the PH→PH group (5.7%) had an EFS event than in the PH→H group (10.3%) or the H→H group (17.4%). Patients in the PH→PH group had a 54% decrease in the risk of an EFS event compared to those treated with single HER2 blockade in the neoadjuvant and adjuvant settings (i.e., H→H; HR = 0.46; 95% CI: 0.26–0.82; Table 3). There was a 15% reduction in EFS event risk in the PH→PH group compared with the PH→H group (HR = 0.85; 95% CI: 0.44–1.65; Table 3).

Since the median follow-up time was different among the studies, the 4-year EFS rates were compared for each treatment modality. Among patients who had a pCR, 4-year EFS was 86% (95% CI: 81–89%) in the H→H group, 90% (95% CI: 85–94%) in the PH→H group, and 95% (95% CI: 92–97%) in the PH→PH group (Table 4). Among patients who had residual disease, 4-year EFS was 64% (95% CI: 59–68%) in the H→H group, 80% (95% CI: 75–85%) in the PH→H group, and 87% (95% CI: 82–91%) in the PH→PH group.

### 3.3. Type of Recurrence

Overall, there was a greater frequency of distant recurrences among patients with residual disease compared to those with a pCR (Table S2). Irrespective of pCR status, recurrences of all types—distant, local, regional, and new contralateral breast cancer—were less frequent with PH→PH compared with H→H or PH→H.

## 4. Discussion

This pooled analysis of the HannaH, NeoSphere, TRYPHAENA, BERENICE, and KRISTINE studies shows that patients who attained a pCR after neoadjuvant HER2-targeted treatment had a better long-term outcome as defined by EFS compared to those with residual disease, regardless of clinical stage, nodal status, hormone receptor status, or treatment modality. Overall, patients with a pCR after neoadjuvant treatment had a 65% reduction in the risk of an EFS event compared to those with residual disease (HR = 0.35; 95% CI: 0.27–0.46).

Our findings are consistent with a pooled analysis of nearly 12,000 patients treated for EBC, demonstrating that patients with a pCR (ypT0/Tis ypN0) had a 52% reduction in the risk of an EFS event at 5 years compared with patients with residual disease [2]. In that pooled analysis, the association between pCR and favorable outcomes was most pronounced in tumor types typically associated with poor prognosis, such as triple-negative breast cancer, in which a 76% reduction in the risk of an EFS event was reported, and HER2-positive breast cancer, in which a 41% reduction in the risk of an EFS event was reported. Our findings are also consistent with a recent pooled analysis of 3710 patients with HER2-positive breast cancer, showing that patients with a pCR after neoadjuvant therapy have longer 5-year survival compared to those with residual disease [6]. Our results extend these observations by evaluating the potential effect of additional baseline factors in patients with HER2-positive breast cancer who are treated with varying chemotherapy and HER2-targeted regimens. A pCR was associated with a substantially decreased risk of an EFS event irrespective of baseline clinical stage and nodal status, as well as hormone receptor status and HER2-targeted regimen.

While patients with a pCR had a decreased risk of an EFS event regardless of HER2-targeted regimen, the magnitude of risk reduction differed among HER2-targeted regimens. The greatest reduction was seen in patients treated with PH in both the neoadjuvant and adjuvant settings. While current treatment guidelines advocate continued HER2-targeted therapy in the adjuvant setting in patients with a pCR [8,10], they generally do not distinguish between single or dual HER2 blockade, likely because these regimens have not been directly compared in this setting in a prospective clinical trial. A recent real-world study of patients with HER2-positive EBC who attained a pCR after treatment with chemotherapy in combination with pertuzumab and trastuzumab in the neoadjuvant setting and who were treated with trastuzumab in the adjuvant setting demonstrated a 90% 4-year EFS rate [26]. These real-world data are consistent with our pooled analysis from randomized clinical trial data highlighting the risk of recurrence in patients who had attained a pCR and who were subsequently treated with trastuzumab alone in the adjuvant setting. Furthermore, while limited, our pooled analysis suggests that a dual HER2-targeted regimen compared with trastuzumab alone in the adjuvant setting is associated with improved long-term outcomes. Ongoing studies (CompassHER2-pCR [NCT04266249] and DESCRESCENDO [NCT04675827]) are currently evaluating chemotherapy de-escalation to four cycles in the neoadjuvant setting with continued adjuvant treatment of pertuzumab and trastuzumab in patients with a pCR. Efforts to optimize therapy will decrease toxicities—which are mostly due to chemotherapy—and improve quality of life.

To optimize treatment decisions, the risk–benefit profile of the treatment must be evaluated. Thus, these efficacy data must also be considered in the context of the safety and tolerability of the treatment regimens. A comprehensive pooled safety analysis is challenging because safety data were not collected and reported in the same way in all studies. Safety data from the individual studies has been extensively reported, and suggest that toxicity is not markedly increased with the addition of pertuzumab except for diarrhea which is higher particularly in the neoadjuvant setting where it is given concurrently with chemotherapy (Table S3; [5,14,15,16,17,18,19,20,21,24]). Docetaxel plus pertuzumab and trastuzumab was compared with docetaxel plus trastuzumab for treatment of HER2-positive EBC in the NEOSPHERE trial [14,17]. The addition of pertuzumab did not result in an increase in grade ≥ 3 adverse events or serious adverse events. However, all-grade diarrhea was increased (46% vs. 34%) [14]. There was also a modest increase in left ventricular ejection fraction (LVEF) decline to <50% and by ≥10 percentage points from baseline throughout the neoadjuvant, adjuvant, and post-treatment follow-up periods with docetaxel plus pertuzumab and trastuzumab compared to docetaxel plus trastuzumab (8% vs. 2%) [17]. All cases resolved to LVEF of ≥50% without intervention. A similar pattern was seen in the phase 3 APHINITY trial, which compared adjuvant chemotherapy plus pertuzumab and trastuzumab with chemotherapy plus placebo and trastuzumab. The incidence of grade ≥3 adverse events was generally similar between groups except for an increase in diarrhea in the pertuzumab group (9.8% vs. 3.7%). However, this increase appeared to be associated with chemotherapy since rates were similar in the post-chemotherapy treatment period (grade ≥ 3 diarrhea 0.5% with pertuzumab plus trastuzumab and 0.2% with placebo plus trastuzumab) [22]. Notably, patient-reported outcome data indicated similar levels of role, social, cognitive, and emotional functioning in both treatment groups, suggesting that patients were able to maintain their functioning even in the context of increased diarrhea [27]. There was also a modest increase in the incidence of New York Heart Association (NYHA) class III or IV heart failure with an LVEF decline to <50% and by ≥10 percentage points from baseline (0.6% with pertuzumab plus trastuzumab and 0.2% with placebo plus trastuzumab) [22].

The BERENICE study evaluated cardiac safety with pertuzumab-plus-trastuzumab-containing regimens and showed a low incidence of cardiac toxicity in the neoadjuvant period. Three patients (1.5%) treated with dose-dense doxorubicin/cyclophosphamide followed by pertuzumab and trastuzumab plus paclitaxel (i.e., Cohort A) had NYHA class III/IV heart failure events, and no patients treated with fluorouracil/epirubicin/cyclophosphamide followed by pertuzumab and trastuzumab plus docetaxel (Cohort B) had such events [19]. Thirteen of the 199 patients (6.5%) in Cohort A and four of the 198 (2.0%) patients in Cohort B had symptomatic and asymptomatic LVEF declines to <50% and by ≥10 percentage points from baseline. The 5-year follow-up data showed a similarly low incidence of cardiac toxicity [24]. These data suggest an acceptable cardiac toxicity profile with the addition of pertuzumab to trastuzumab and these chemotherapy regimens, and thus support the safety of continuation of pertuzumab plus trastuzumab from the neoadjuvant to adjuvant setting.

While our analysis demonstrated improved outcomes over time as the standard of care has evolved, patients with a pCR still experienced disease recurrence, underlining the need to provide standard of care to all patients and for further research to better identify prognostic factors for recurrence. Outcomes between our analysis and the APHINITY trial cannot be directly compared since APHINITY evaluated patients in the adjuvant setting without consideration of possible neoadjuvant regimens. APHINITY did show that outcomes with pertuzumab plus trastuzumab were substantially improved compared with those observed in the early adjuvant studies with single-agent trastuzumab [23]. While in favor of combined antibody treatment, the magnitude of the critical efficacy differences (EFS/survival) are modest even with longer follow-up. However, patients with lymph node–positive disease had a 4.5 percentage point improvement in IDFS with the addition of pertuzumab (6-year IDFS rate, 88% vs. 83%), but IDFS rates were similar in patients with lymph node–negative disease (6-year IDFS rate, 95% in both arms) [23].

A limitation of this pooled analysis, as noted, is that there were a larger number of patients with clinical stage II disease and hormone receptor-positive disease at baseline in the PH→PH group compared with the other treatment groups. In addition, the H→H and PH→H groups had a higher number of patients with T4 lesions, which could have affected the results. However, potential confounding by imbalances in clinical stage and hormone receptor status among the treatment groups was addressed by using the IPTW approach. A known limitation of neoadjuvant studies is the potential for clinically negative lymph nodes to be positive at the time of surgery [28]. A recent analysis showed that among patients who were cN0 pretreatment, the ypN0 rate was 89%, indicating that ≥10% of patients were node-positive at diagnosis [28]. Additionally, per protocol in the NeoSphere study, patients received part of the chemotherapy backbone (fluorouracil, epirubicin, cyclophosphamide) after surgery, which could have contributed to the lower pCR rates compared with other studies in which all of the chemotherapy was administered before surgery. It should also be noted that the chemotherapy backbones—including treatment with anthracyclines—differed among the studies, and the contribution of this to the overall results is not known. Finally, approximately a third of the patients in the PH→PH group were from the KRISTINE study, which had the shortest follow-up time (36 months vs. >60 months in the other studies).

## 5. Conclusions

In this pooled analysis, patients with HER2-positive EBC who had a pCR after neoadjuvant systemic chemotherapy plus HER2-directed therapy had a reduced risk of recurrence compared with patients with residual disease. However, recurrences still occurred, supporting continued HER2-targeted therapy as standard of care in this setting. Clinical benefit appeared the greatest when treatment included pertuzumab and trastuzumab in both the neoadjuvant and adjuvant settings, with the lowest recurrence rates observed with this combination.

## Figures and Tables

**Table 1:** Baseline characteristics.

Characteristic, n (%)	Overall (n = 1763)	H→H (n = 703)	PH→H (n = 439)	PH→PH (n = 621)
Age (years)				
<40	314 (17.8)	115 (16.4)	73 (16.6)	126 (20.3)
40−60	1302 (73.9)	531 (75.5)	336 (76.5)	435 (70.0)
>60	147 (8.3)	57 (8.1)	30 (6.8)	60 (9.7)
Clinical stage				
I	22 (1.2)	21 (3.0)	0	1 (0.2)
II	933 (52.9)	285 (40.5)	204 (46.5)	444 (71.5)
III	807 (45.8)	397 (56.5)	235 (53.5)	175 (28.2)
Unknown	1 (0.1)	0	0	1 (0.2)
Hormone receptor status				
Negative	783 (44.4)	328 (46.7)	223 (50.8)	232 (37.4)
Positive	963 (54.6)	372 (52.9)	215 (49.0)	376 (60.5)
Unknown	17 (1.0)	3 (0.4)	1 (0.2)	13 (2.1)
Tumor stage at study entry				
T1	72 (4.1)	43 (6.1)	0	29 (4.7)
T2	926 (52.5)	304 (43.2)	201 (45.8)	421 (67.8)
T3	406 (23.0)	138 (19.6)	144 (32.8)	124 (20.0)
T4	354 (20.1)	217 (30.9)	91 (20.7)	46 (7.4)
Unknown	5 (0.3)	1 (0.1)	3 (0.7)	1 (0.2)
Clinical nodal stage at study entry				
Node-positive	536 (30.4)	164 (23.3)	128 (29.2)	244 (39.3)
Node-negative	1206 (68.4)	538 (76.5)	308 (70.2)	360 (58.0)
Unknown	21 (1.2)	1 (0.1)	3 (0.7)	17 (2.7)

Abbreviations: H→H, trastuzumab in the neoadjuvant setting followed by trastuzumab in the adjuvant setting; PH→H, pertuzumab plus trastuzumab in the neoadjuvant setting followed by trastuzumab in the adjuvant setting; PH→PH, pertuzumab plus trastuzumab in the neoadjuvant setting followed by pertuzumab plus trastuzumab in the adjuvant setting.

**Table 2:** pCR by baseline tumor and clinical stage and HER2-targeted treatment modality.

n (%)	pCR	Residual Disease	Total
Tumor stage			
T1	36 (50.0)	36 (50.0)	72
T2	447 (48.3)	479 (51.7)	926
T3	177 (43.6)	229 (56.4)	406
T4	111 (31.4)	243 (68.6)	354
Unknown	2 (40.0)	3 (60.0)	5
Clinical stage			
0	0 (0)	1 (100.0)	1
I	11 (50.0)	11 (50.0)	22
II	456 (48.9)	477 (51.1)	933
III	306 (37.9)	501 (62.1)	807
Hormone receptor status			
Negative	434 (55.4)	349 (44.6)	783
Positive	331 (34.4)	632 (65.6)	963
Unknown	8 (47.1)	9 (52.9)	17
Treatment modality			
H→H	236 (33.6)	467 (66.4)	703
PH→H	185 (42.1)	254 (57.9)	439
PH→PH	352 (56.7)	269 (43.3)	621

Abbreviations: HER2, human epidermal growth factor receptor 2; pCR, pathological complete response.

**Table 3:** Treatment effect by pathological complete response and treatment modality using inverse probability of treatment weighting analysis *.

	**All Patients, Regardless of Pathological Complete Response Status**					
	**H→H (n = 703)**		**PH→H (n = 439)**		**PH→PH (n = 621)**	
Patients with EFS event, %	217 (30.9)		79 (18.0)		53 (8.5)	
Patients without EFS event, %	486 (69.1)		360 (82.0)		568 (91.5)	
Hazard ratio versus H→H (95% CI)			0.56 (0.43–0.73)		0.36 (0.26–0.49)	
Hazard ratio versus PH→H (95% CI)					0.67 (0.47–0.96)	
	**pCR**			**Residual Disease**		
	**H→H** **(n = 236)**	**PH→H** **(n = 185)**	**PH→PH** **(n = 352)**	**H→H** **(n = 467)**	**PH→H** **(n = 254)**	**PH→PH** **(n = 269)**
Patients with EFS event, %	41 (17.4)	19 (10.3)	20 (5.7)	176 (37.7)	60 (23.6)	33 (12.3)
Patients without EFS event, %	195 (82.6)	166 (89.7)	332 (94.3)	291 (62.3)	194 (76.4)	236 (87.7)
Time to event (months)						
Hazard ratio versus H→H (95% CI)		0.59 (0.34–1.01)	0.46 (0.26–0.82)		0.60 (0.45–0.81)	0.43 (0.30–0.63)
Hazard ratio versus PH→H (95% CI)		0.85 (0.44–1.65)			0.75 (0.49–1.16)	

Abbreviations: EFS, event-free survival. * Adjusted for hormone receptor status (positive, negative); clinical stage (I, II, III); and age group (<40, 40–65, >65 years).

**Table 4:** Four-year event-free survival rate in patients with and without a pathological complete response by treatment modality using inverse probability of treatment weighting analysis *.

	**4-Year Event-Free Survival Rate in Patients with pCR**		
	**H→H** **(n = 236)**	**PH→H** **(n = 185)**	**PH→PH** **(n = 352)**
Patients remaining at risk, n	179	155	219
4-year event-free survival rate, % (95% CI)	86 (81–89)	90 (85–94)	95 (92–97)
	**4-Year Event-Free Survival Rate in Patients with** **Residual Disease**		
	**H→H** **(n = 467)**	**PH→H** **(n = 254)**	**PH→PH** **(n = 269)**
Patients remaining at risk, n	251	176	107
4-year event-free survival rate, % (95% CI)	64 (59–68)	80 (75–85)	87 (82–91)

* Adjusted for hormone receptor status (positive, negative), clinical stage (I, II, III), and age group (<40, 40–65, >65 years).

## Data Availability

Qualified researchers may request access to individual patient level data through the clinical study data request platform (https://vivli.org/, accessed on 26 August 2022). Further details on Roche’s criteria for eligible studies are available here (https://vivli.org/members/ourmembers/, accessed on 26 August 2022). For further details on Roche’s Global Policy on the Sharing of Clinical Information and how to request access to related clinical study documents, see here (https://www.roche.com/research_and_development/who_we_are_how_we_work/clinical_trials/our_commitment_to_data_sharing.htm, accessed on 26 August 2022).”Roche’s Data Sharing Statement2As of 5th May, Roche is sharing data through the Vivli data sharing platform. This is reflected in the above data sharing statement.

## Supplementary Materials

The following supporting information can be downloaded at https://www.mdpi.com/article/10.3390/cancers14205051/s1, Figure S1: Balance plots depicting the absolute standardized mean differences in key baseline characteristics before and after adjusting the analysis using the inverse probability of treatment weighting method; Table S1: Data sources; Table S2: Types of recurrence in patients with and without a pathological complete response; Table S3: Key safety data by study.
