# Supplementary material for: Event-Free Survival in Patients with Early HER2-Positive Breast Cancer with a Pathological Complete Response after HER2-Targeted Therapy: A Pooled Analysis
Source: Cancers (Basel). 2022 Oct 15;14(20):5051. doi: 10.3390/cancers14205051 (PMC9599862; doi:10.3390/cancers14205051)
Supplement: Supplementary file 1 [file cancers-14-05051-s001.zip › cancers-1910914-SI.pdf]

Supplementary Material

Event-Free Survival in Patients with Early HER2-Positive Breast Cancer with a Pathological Complete Response after HER2-Targeted Therapy: A Pooled Analysis

Sandra M. Swain [1],*, Harrison Macharia [2], Javier Cortes [3,4], Chau Dang [5], Luca Gianni [6], Sara A. Hurvitz [7], Christian Jackisch [8], Andreas Schneeweiss [9], Dennis Slamon [7], Pinuccia Valagussa [6], Yolande du Toit [10], Dominik Heinzmann [2], Adam Knott [2], Chunyan Song [10] and Patricia Cortazar [10]

1 Lombardi Comprehensive Cancer Center, Georgetown University Medical Center, MedStar Health, Washington, DC 20057, USA
2 F. Hoffmann-La Roche Ltd., 4070 Basel, Switzerland
3 Quirónsalud Group, IOB Institute of Oncology, Madrid and Barcelona, 08023 Barcelona, Spain
4 Vall d'Hebron Institute of Oncology (VHIO), 08023 Barcelona, Spain
5 Department of Medicine, Breast Medicine Service, Memorial Sloan Kettering Cancer Center, New York, NY 10013, USA
6 Fondazione Michelangelo, 20121 Milano, Italy
7 David Geffen School of Medicine, University of California Los Angeles, Los Angeles, CA 94720, USA
8 Sana Klinikum Offenbach, 63069 Offenbach, Germany
9 National Center for Tumor Diseases (NCT), 69120 Heidelberg, Germany
10 Genentech, Inc., South San Francisco, CA 94080, USA
* Correspondence: sandra.swain@georgetown.edu; Tel.: +1-202-687-8487

Contents

Table S1. 1
Figure S1. 4
Table S2. 5
Table S3. 6

Table S1. Data sources. The arrows indicate the time of surgery such that treatments to the left of the arrow were given prior to surgery (i.e., neoadjuvant setting) and treatments to the right of the arrow were given after surgery (i.e., adjuvant setting)

**Study (enrollment period)**	**Treatment Arms**	**Patients (*n*)**	**Treatment Modality**	**Median Duration of Follow-up (months)**
HannaH (10/09 to 12/10)	1: Docetaxel + trastuzumab SC (4 cycles) followed by FEC + trastuzumab SC (4 cycles) [surgery] trastuzumab SC Q3W to 1 year	297	H→H	71.8
	2: Docetaxel trastuzumab IV (4 cycles) followed by FEC + trastuzumab IV (4 cycles) [surgery] trastuzumab IV Q3W to 1 year	299	H→H	
NeoSphere (12/07 to 12/09)	1: Docetaxel + trastuzumab (4 cycles) [surgery] FEC + trastuzumab (3 cycles), followed by trastuzumab Q3W to 1 year	107	H→H	60.9
	2: Docetaxel + trastuzumab + pertuzumab (4 cycles) [surgery] FEC + trastuzumab (3 cycles), followed by trastuzumab Q3W to 1 year	107	PH→H	
	3: Trastuzumab + pertuzumab (4 cycles) [surgery] docetaxel (4 cycles) followed by FEC + trastuzumab (3 cycles), followed by trastuzumab Q3W for 1 year	107	PH→H	

TRYPHAENA (12/09 to 1/11)	1: FEC + trastuzumab + pertuzumab (3 cycles) followed by docetaxel + trastuzumab + pertuzumab (3 cycles) [surgery] trastuzumab Q3W to 1 year	73	PH→H	61.4
	2: FEC (3 cycles) followed by docetaxel + trastuzumab + pertuzumab (3 cycles) [surgery] trastuzumab Q3W to 1 year	75	PH→H	
	3: Docetaxel + carboplatin + trastuzumab + pertuzumab (6 cycles) [surgery] trastuzumab Q3W to 1 year	77	PH→H	
BERENICE (7/14 to 8/15)	1: ddAC (4 cycles) followed by paclitaxel (weekly for 12 weeks) and trastuzumab + pertuzumab (Q3W for 4 cycles) [surgery] trastuzumab + pertuzumab Q3W to 1 year	199	PH→PH	64.5
	2: FEC (4 cycles) followed by docetaxel + trastuzumab + pertuzumab (4 cycles) [surgery] trastuzumab + pertuzumab Q3W to 1 year	201	PH→PH	
KRISTINE (6/14 to 6/15)	1: Docetaxel + carboplatin + trastuzumab + pertuzumab (6 cycles) [surgery] trastuzumab + pertuzumab (12 cycles)	221	PH→PH	36.9

Abbreviations: ddAC, dose-dense doxorubicin and cyclophosphamide; FEC, 5-fluorouracil, epirubicin, and cyclophosphamide; H→H, trastuzumab in the neoadjuvant setting followed by trastuzumab in the adjuvant setting; IV, intravenous; PH→H, pertuzumab plus trastuzumab in the neoadjuvant setting

followed by trastuzumab in the adjuvant setting; PH→PH, pertuzumab plus trastuzumab in the neoadjuvant setting followed by pertuzumab plus trastuzumab in the adjuvant setting; Q3W, once every 3 weeks; SC, subcutaneous.

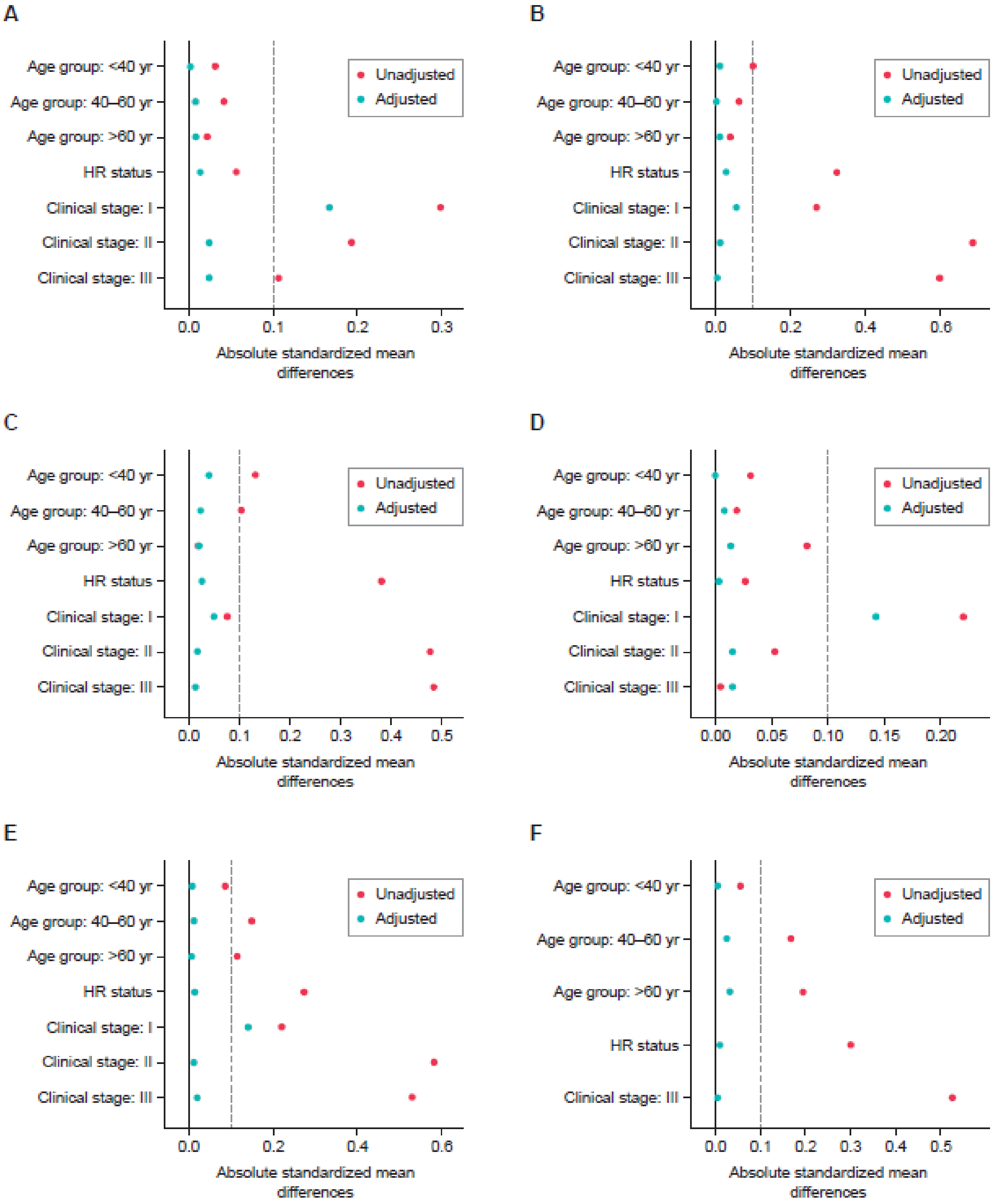

Figure S1. Balance plots depicting the absolute standardized mean differences in key baseline characteristics before and after adjusting the analysis using the inverse probability of treatment weighting method. (A) Patients with a pCR in the PH→H vs. H→H groups; (B) patients with a pCR in the PH→PH vs. H→H groups; (C) patients with a pCR in the PH→PH vs. PH→H groups; (D) patients with residual disease in the PH→H vs. H→H groups; (E) patients with residual disease in the PH→PH vs. H→H groups; and (F) patients with residual disease in the PH→PH vs. PH→H groups

Table S2. Types of recurrence in patients with and without a pathological complete response

	**pCR**			**Residual Disease**		
***n* (%)**	**H→H (*n* = 236)**	**PH→H (*n* = 185)**	**PH→PH (*n* = 352)**	**H→H (*n* = 467)**	**PH→H (*n* = 254)**	**PH→PH (*n* = 269)**
Overall	43 (18.2)	17 (9.2)	16 (4.5)	149 (31.9)	65 (25.6)	28 (10.4)
Distant	26 (11.0)	10 (5.4)	12 (3.4)	97 (20.8)	37 (14.6)	16 (5.9)
Local	12 (5.1)	5 (2.7)	1 (0.3)	24 (5.1)	18 (7.1)	6 (2.2)
Regional	3 (1.3)	2 (1.1)	2 (0.6)	13 (2.8)	8 (3.1)	4 (1.5)
New contralateral breast cancer	2 (0.8)	0	1 (0.3)	15 (3.2)	2 (0.8)	2 (0.7)

Abbreviations: H→H, trastuzumab in the neoadjuvant setting followed by trastuzumab in the adjuvant setting; pCR, pathological complete response; PH→H, pertuzumab plus trastuzumab in the neoadjuvant setting followed by trastuzumab in the adjuvant setting; PH→PH, pertuzumab plus trastuzumab in the neoadjuvant setting followed by pertuzumab plus trastuzumab in the adjuvant setting.

Table S3. Key safety data by study

Data for NEOSPHERE, TRYPHAENA, and BERENICE are from the latest pertuzumab label. Data from HANNAH are from the primary publication (Ismael G, et al, Lancet Oncol. 2012;13:869–878) as are data from KRISTINE (Hurvitz SA, et al, J Clin Oncol. 2019;37:2206–2216) since these studies are not included in the pertuzumab label.

	**H→H**<br>**HANNAH**	**PH→H**<br>**NEOSPHERE**	**PH→H**<br>**TRYPHAENA**	**PH→PH**<br>**BERENICE**	**PH→PH**<br>**KRISTINE**
**Patients with grade ≥3 diarrhea**	**Data are from neoadjuvant and adjuvant phases combined**<br>**H (IV)**<br>**(n = 298)**<br>3%<br>**H (SC)**<br>**(n = 297)**<br>3%	**Data are from neoadjuvant and adjuvant phases combined**<br>**P + H + T**<br>**(n = 107)**<br>6%<br>**P + H**<br>**(n = 108)**<br>0	**Data are from neoadjuvant and adjuvant phases combined**<br>**FEC + H + P → T + H + P**<br>**(n = 72)**<br>4%<br>**FEC→ T + H + P**<br>**(n = 75)**<br>5%<br>**TCH + P**<br>**(n = 76)**<br>12%	**Data are from neoadjuvant and adjuvant phases combined**<br>**Cohort A:**<br>**ddAC→PacPH**<br>**(n = 199)**<br>3%<br>**Cohort B:**<br>**FEC→TPH**<br>**(n = 198)**<br>10%	**TCH + P→PH**<br>**(n = 219)**<br>**NEOADJUVANT**<br>15%<br>**ADJUVANT**<br>1%

**Cardiac safety**	**H→H**<br><br>**HANNAH**	**PH→H**<br><br>**NEOSPHERE**	**PH→H**<br><br>**TRYPHAENA**	**PH→PH**<br><br>**BERENICE**	**PH→PH**<br><br>**KRISTINE**
	**Data are from neoadjuvant and adjuvant phases combined**	**Data are from the neoadjuvant phase**	**Data are from neoadjuvant and adjuvant phases combined**	**Data are from the neoadjuvant phase**	**Data are from neoadjuvant and adjuvant phases combined**
**Patients with LVEF decline to <50% and by ≥10 percentage points from baseline**	**H (IV)**<br><br>**(n = 298)**<br><br>2%<br><br>**H (SC)**<br><br>**(n = 297)**<br><br>2%	**P + H + T**<br><br>**(n = 107)**<br><br>8%	**FEC + H + P → T + H + P**<br><br>**(n = 72)**<br><br>7%<br><br>**FEC→ T + H + P**<br><br>**(n = 75)**<br><br>16%<br><br>**TCH + P**<br><br>**(n= 76)**<br><br>11%	**Cohort A:**<br><br>**ddAC→PacPH**<br><br>**(n = 199)**<br><br>7%<br><br>**Cohort B:**<br><br>**FEC→TPH**<br><br>**(n = 198)**<br><br>2%	**TCH + P→PH**<br><br>**(n = 219)**<br><br>2%
**Patients with left ventricular**	**1%**	**P + H + T**<br><br>**(n = 107)**	**FEC + H + P → T + H + P**	**Cohort A:**<br><br>**ddAC→PacPH**	**Not reported**

**dysfunction**		3%	**(n = 72)**<br>6%<br>**FEC→ T + H + P**<br>**(n = 75)**<br>4%<br>**TCH + P**<br>**(n= 76)**<br>3%	**(n = 199)**<br>7%<br>**Cohort B:**<br>**FEC→TPH**<br>**(n = 198)**<br>4%	
**Patients with symptomatic left ventricular dysfunction (NYHA class III/IV congestive heart failure)**	**Class III**<br>0<br>**Class IV**<br>0	**P + H + T**<br>**(n = 107)**<br>0<br>**P + H**<br>**(n = 108)**<br>1%	**FEC + H + P → T + H + P**<br>**(n = 72)**<br>0<br>**FEC→ T + H + P**<br>**(n = 75)**<br>4%<br>**TCH + P**<br>**(n= 76)**<br>1%	**Cohort A:**<br>**ddAC→PacPH**<br>**(n = 199)**<br>2%<br>**Cohort B:**<br>**FEC→TPH**<br>**(n = 198)**<br>0	**TCH + P→PH**<br>**(n = 219)**<br>**1%**

Abbreviations: ddAC, dose-dense doxorubicin and cyclophosphamide; FEC, 5-fluorouracil, epirubicin, and cyclophosphamide; H, trastuzumab; H→H, trastuzumab in the neoadjuvant setting followed by trastuzumab in the adjuvant setting; IV, intravenous; LVSD, left ventricular systolic dysfunction; P,

pertuzumab; Pac, paclitaxel; PH→H, pertuzumab plus trastuzumab in the neoadjuvant setting followed by trastuzumab in the adjuvant setting; PH→PH, pertuzumab plus trastuzumab in the neoadjuvant setting followed by pertuzumab plus trastuzumab in the adjuvant setting; SC, subcutaneous, T, docetaxel; TCH, docetaxel + carboplatin + trastuzumab.
